# Bedside patent ductus arteriosus ligation via anterior mini-thoracotomy in preterm infants weighing ≤ 1500 g: a retrospective cohort study

**DOI:** 10.1186/s13019-026-04581-0

**Published:** 2026-07-17

**Authors:** Serkan Secici, Seyyit Bahaettin Oncu, Serkan Terkanlıoglu

**Affiliations:** 1 Department of Cardiovascular Surgery, Medicana Bursa Hospital, Bursa, Turkey; 2 Present Address: Department of Cardiovascular Surgery, Manisa Celal Bayar University Faculty of Medicine, Manisa, Turkey; 3 Department of Pediatric Cardiology, Medicana Bursa Hospital, Bursa, Turkey; 4 Department of Anesthesiology and Reanimation, Medicana Bursa Hospital, Bursa, Turkey

**Keywords:** Patent ductus arteriosus, Preterm infants, Bedside surgery, Neonatal intensive care unit, Mini-thoracotomy, Congenital cardiac surgery

## Abstract

**Background:**

Hemodynamically significant patent ductus arteriosus (hsPDA) remains a major clinical challenge in preterm infants. Surgical ligation remains necessary in selected patients when medical treatment fails or is contraindicated, or when transcatheter closure is not feasible. Left anterior mini-thoracotomy (LAMT) has been proposed as a less invasive alternative, and bedside ligation in the neonatal intensive care unit (NICU) may avoid transport-related risks in critically ill neonates.

**Methods:**

This retrospective cohort study included preterm infants weighing ≤ 1500 g who underwent surgical PDA ligation between August 2020 and May 2025. Patients were grouped according to the location of surgery: bedside ligation in the NICU or ligation in the operating room (OR). All procedures were performed by a single surgeon using a standardized LAMT. Demographic characteristics, perioperative variables, postoperative outcomes, and mortality were compared between groups. Continuous variables were analyzed using the Mann–Whitney U test, and categorical variables using Fisher’s exact test.

**Results:**

Sixty-eight preterm infants were included (NICU group, *n* = 25; OR group, *n* = 43). Baseline demographic characteristics and preoperative comorbidities were largely comparable between groups. All procedures were completed without conversion to sternotomy, intraoperative mortality, major bleeding, or vascular injury. The duration of postoperative mechanical ventilation, postoperative NICU stay, and postmenstrual age at NICU discharge did not differ significantly between groups. Two deaths occurred in the NICU group from progressive multiorgan failure on postoperative days 11 and 15. Both weighed less than 1000 g, had preoperative acute renal dysfunction, and required vasoactive support before surgery. Neither death was associated with an intraoperative complication. No surgical site infection or postoperative sepsis was documented in either group within the first 7 postoperative days.

**Conclusions:**

Bedside PDA ligation via LAMT was technically feasible in selected preterm infants weighing ≤ 1500 g, and all procedures were completed as planned. However, the small sample size and differences in referral status between groups limit definitive conclusions regarding comparative safety.

**Trial registration:**

Not applicable. This study is a retrospective observational study and did not require trial registration.

## Introduction

Patent ductus arteriosus (PDA) is common in preterm infants, particularly at lower gestational ages. It occurs in approximately 30% of infants born before 30 weeks of gestation and up to 60% of those born before 28 weeks [1, 2]. Hemodynamically significant PDA (hsPDA) is associated with respiratory morbidity, feeding intolerance, bronchopulmonary dysplasia, necrotizing enterocolitis, intraventricular hemorrhage, and impaired renal perfusion [3–5].

Pharmacological closure is generally used as the first-line approach. Transcatheter or surgical closure may be considered when pharmacological treatment is unsuccessful or contraindicated [4, 5]. Although transcatheter closure is increasingly used, surgical ligation remains an important option when catheter-based treatment is not feasible or available. It provides immediate and definitive ductal closure but may be associated with bleeding, pneumothorax, chylothorax, diaphragmatic or vocal cord paralysis, post-ligation cardiac syndrome, and long-term chest wall or spinal deformities [6–10].

Left anterior mini-thoracotomy (LAMT) has been described as a less invasive alternative to conventional posterolateral thoracotomy. The limited parasternal incision provides direct access to the pulmonary artery and ductus while reducing the need for lung retraction and extensive tissue dissection [11]. This technique is effective and safe for very low birth weight premature infants, reducing adverse effects, particularly due to decreased operation time and limited intraoperative compressions and lesions of the left lung.

In addition to the surgical approach, the location of the procedure is an important consideration in these fragile infants. Bedside PDA ligation in the neonatal intensive care unit (NICU) may avoid the thermal, respiratory, and hemodynamic risks associated with transport to the operating room (OR). Previous studies have compared bedside and OR ligation and reported acceptable short-term outcomes in both settings [12–14]. However, the surgical techniques differed, and data on the use of a standardized LAMT approach in both settings remain limited.

The present study aimed to compare the perioperative and short-term outcomes of bedside and OR PDA ligation performed using the same standardized LAMT technique in preterm infants weighing ≤ 1500 g.

## Methods

### Study design and patient population

This retrospective cohort study included all consecutive preterm infants weighing ≤ 1500 g at the time of surgery who underwent surgical PDA ligation between August 2020 and May 2025. Infants were grouped according to procedure location: bedside ligation in the NICU or ligation in the OR. All infants in the NICU group were inborn, whereas all infants in the OR group were outborn and referred from external centers. Thus, the study groups reflected referral status rather than random allocation.

The decision for surgical closure was made by the neonatal and pediatric cardiology teams after clinical and echocardiographic assessment of hemodynamic significance using the McNamara staging approach [15]. Surgery was performed when pharmacological treatment failed or was contraindicated. Transcatheter PDA closure was available at our institution but was not performed in preterm infants during the study period.

All infants were already intubated and receiving invasive mechanical ventilation before surgery. Airway and ventilator management were provided by the neonatal intensive care team during bedside procedures and by the anesthesia team in the OR. Infants referred from external centers were transferred back to their referring hospitals once they were clinically stable and no surgical complication had been identified. No predefined transfer criteria were used.

### Perioperative management

For bedside PDA ligation, infants remained in their incubators throughout the procedure. All personnel in the designated surgical area were required to wear operating room attire, including surgical caps and masks. Surgical instruments and equipment were transported from the operating room to the NICU.

In the NICU group, anesthesia was maintained with intravenous fentanyl without volatile anesthetic agents. In the OR group, low-dose volatile anesthesia was combined with intravenous fentanyl. Rocuronium was used for neuromuscular blockade in both groups. Standard monitoring included three-lead electrocardiography, pulse oximetry, noninvasive blood pressure measurement, and body temperature monitoring. Central venous catheterization and invasive arterial monitoring were not performed.

### Surgical technique

**Fig. 1:**
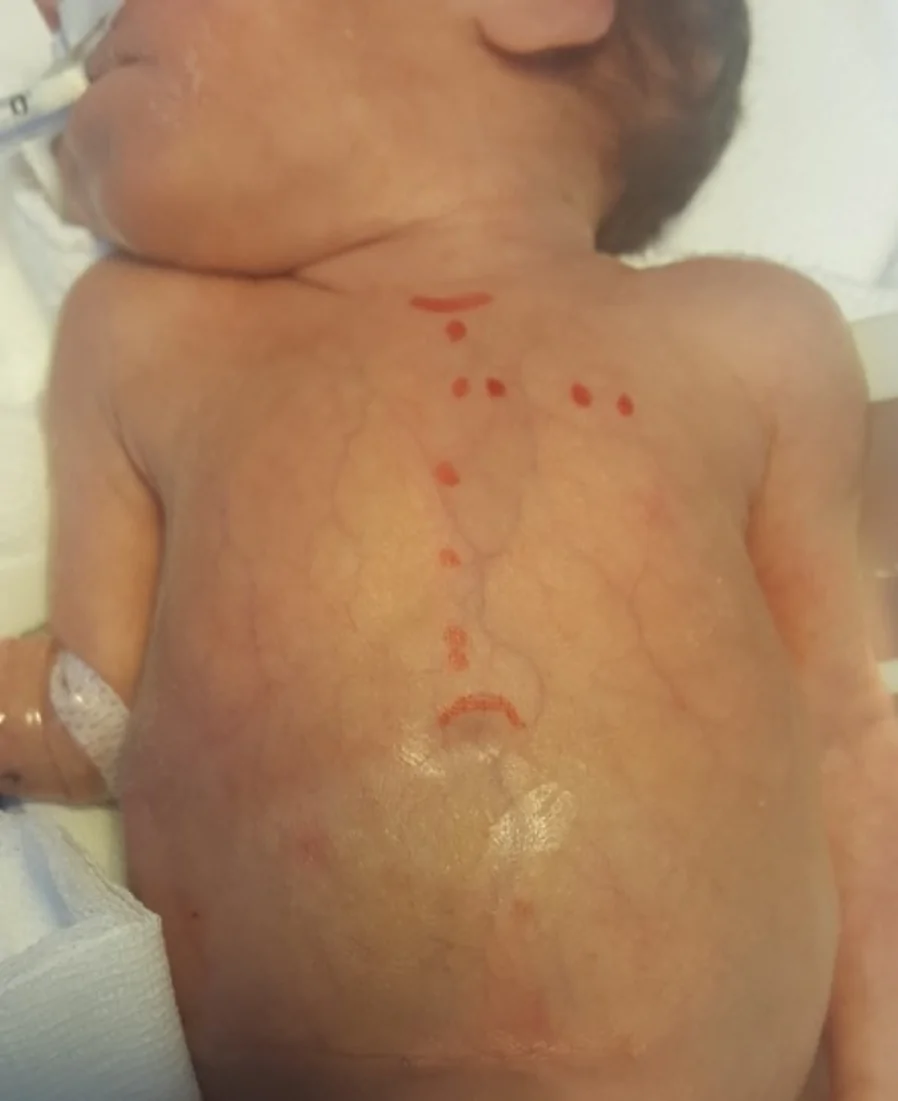
Preoperative skin markings. The short horizontal line in the second left intercostal space indicates the planned left anterior mini-thoracotomy incision. The dotted vertical line along the sternum marks the site of a possible emergency median sternotomy

**Fig. 2:**
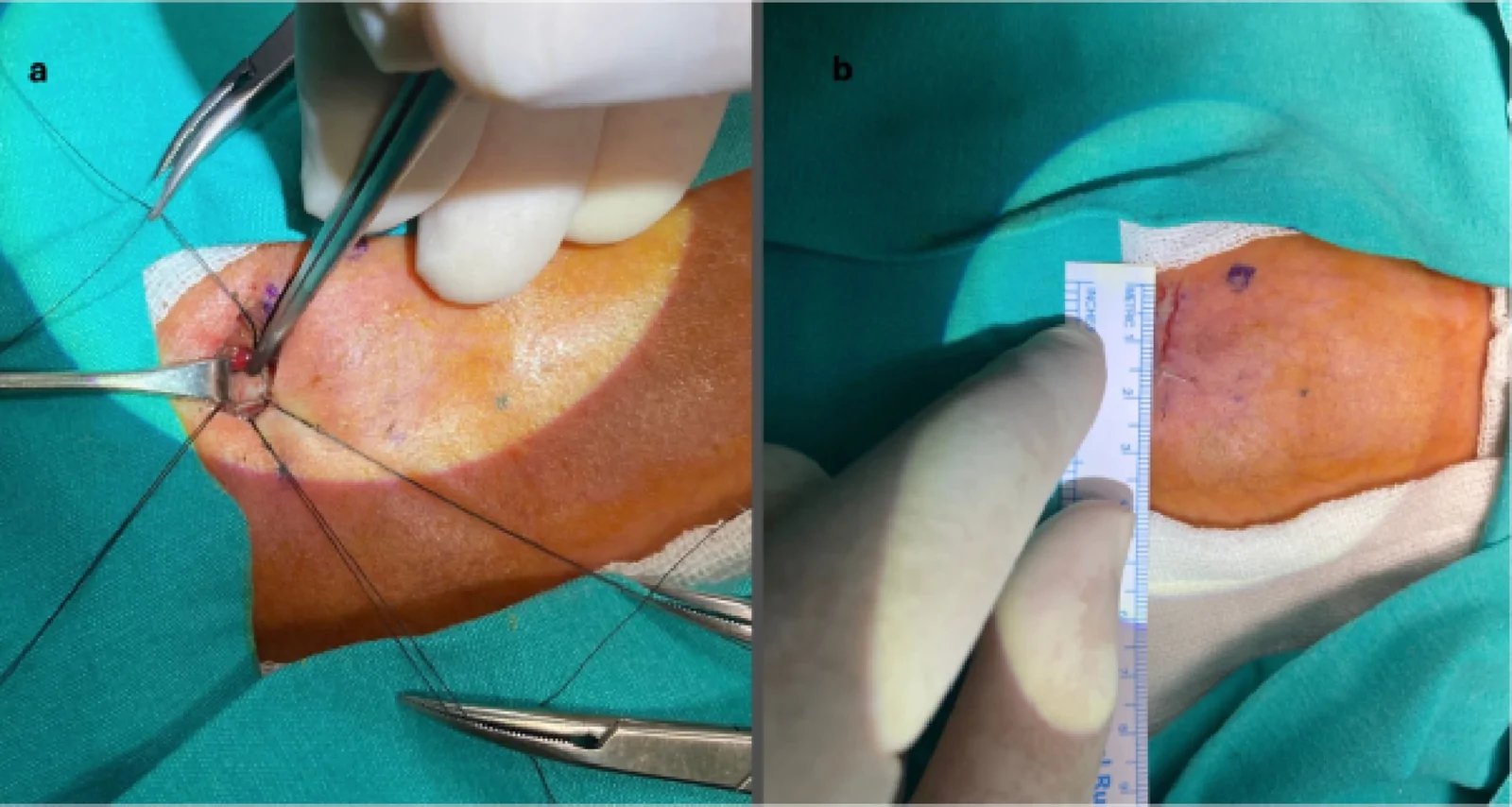
Intraoperative views (**a**) Direct access to the pulmonary artery and PDA without lung retraction; (**b**) Postoperative view of the closed skin incision

All procedures were performed by a single surgeon using a standardized LAMT. With the patient in the supine position, a 1–1.5-cm horizontal parasternal incision was made in the second left intercostal space (Fig. 1). The pericardium was opened longitudinally and suspended to expose the ductus without lung retraction (Fig. 2). The left and right pulmonary arteries and the ductus arteriosus were identified, and the ductus was occluded with one or two metallic vascular clips.

### Data collection and definitions

Baseline and postoperative data were obtained from the medical records. The early postoperative period was defined as the first 7 days after surgery. Postoperative NICU stay was calculated from the day of surgery to discharge from the NICU. Postmenstrual age at NICU discharge was calculated using postmenstrual age at surgery and postoperative NICU stay.

Preoperative acute renal dysfunction was defined according to the modified neonatal KDIGO criteria, based on changes in serum creatinine and urine output [16]. Post-ligation cardiac syndrome was defined as new or increased cardiovascular support accompanied by otherwise unexplained worsening of oxygenation or ventilation within 24 h after ductal closure [8]. The timing and documented cause of each death were reviewed to assess any relation to the surgical procedure.

### Statistical analysis

Continuous variables were presented as median (interquartile range) and compared using the Mann–Whitney U test. Categorical variables were presented as number (percentage) and compared using Fisher’s exact test. Statistical comparisons were not performed for outcomes with no events in either group. A two-sided p-value < 0.05 was considered statistically significant. Multivariable analysis was not performed because of the limited number of outcome events and the complete overlap between procedure location and inborn/outborn status. Analyses were performed using IBM SPSS Statistics version 21.0 (IBM Corp., Armonk, NY, USA).

### Ethical considerations

The study was approved by the Manisa Celal Bayar University Health Sciences Ethics Committee on September 3, 2025 (approval no. 20.478.486/3384). The requirement for informed consent was waived because of the retrospective study design.

## Results

During the study period, 89 infants underwent surgical PDA ligation. Of these, 68 preterm infants weighing ≤ 1500 g at surgery met the inclusion criteria: 25 underwent bedside ligation in the NICU and 43 underwent ligation in the OR. All infants in the NICU group were inborn, whereas all infants in the OR group were outborn. All were receiving invasive mechanical ventilation before surgery. All infants except two had received pharmacological treatment for PDA. The two untreated infants had preoperative acute renal dysfunction.

Baseline characteristics are presented in Table 1. Birth weight, weight at surgery, postnatal age, and PMA at surgery did not differ significantly between groups. Preoperative acute renal dysfunction was present in two infants in the NICU group and none in the OR group. These two infants were the only patients who required preoperative vasoactive support.

All procedures were completed using the planned LAMT approach. No conversion to sternotomy, intraoperative death, major bleeding, or vascular injury occurred. Postoperative outcomes are shown in Table 2. Two infants in the OR group developed respiratory acidosis after transfer back to their referring hospitals. Neither developed hypotension or required vasoactive support, and both improved after adjustment of ventilator settings. One infant in each group developed diaphragmatic paralysis and underwent diaphragmatic plication. One infant in the OR group developed chylothorax. No clinically documented PLCS, surgical site infection, or postoperative sepsis within 7 days was identified in either group. No hypothermia or hypoglycemia was documented at the end of the procedure.

**Fig. 3:**
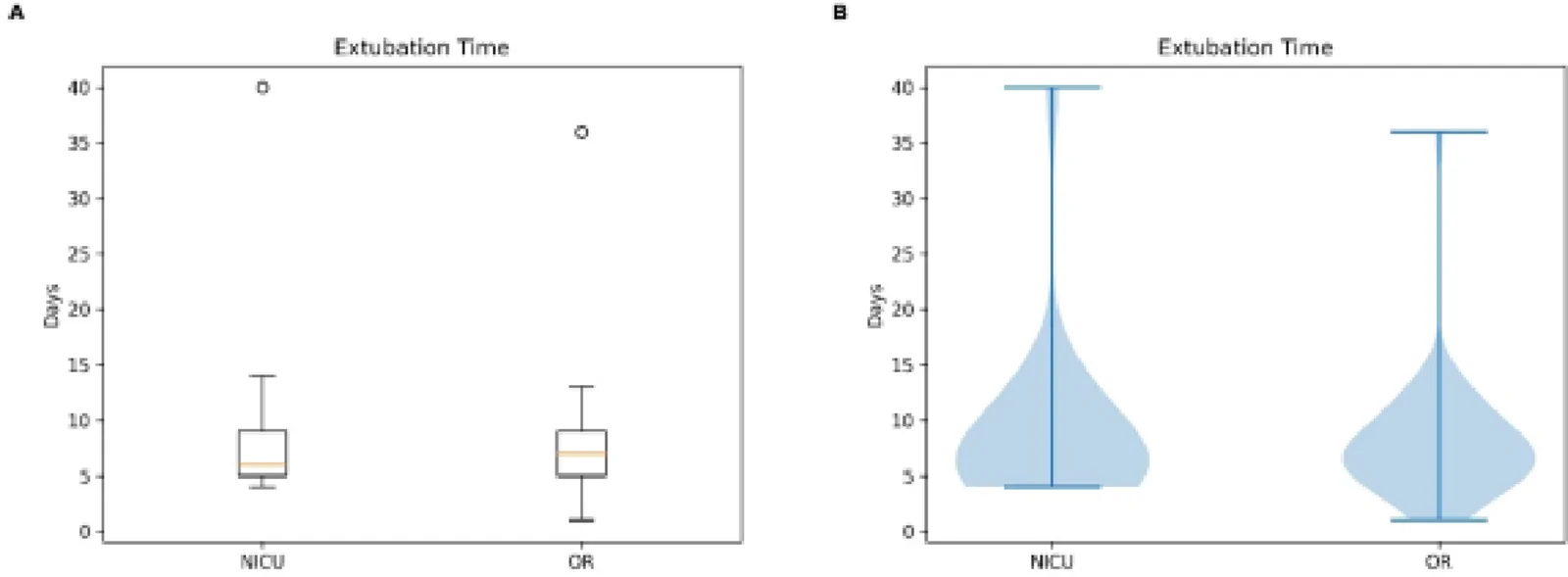
Comparison of postoperative extubation time **(A)** Boxplot **(B)** Violin plot

**Fig. 4:**
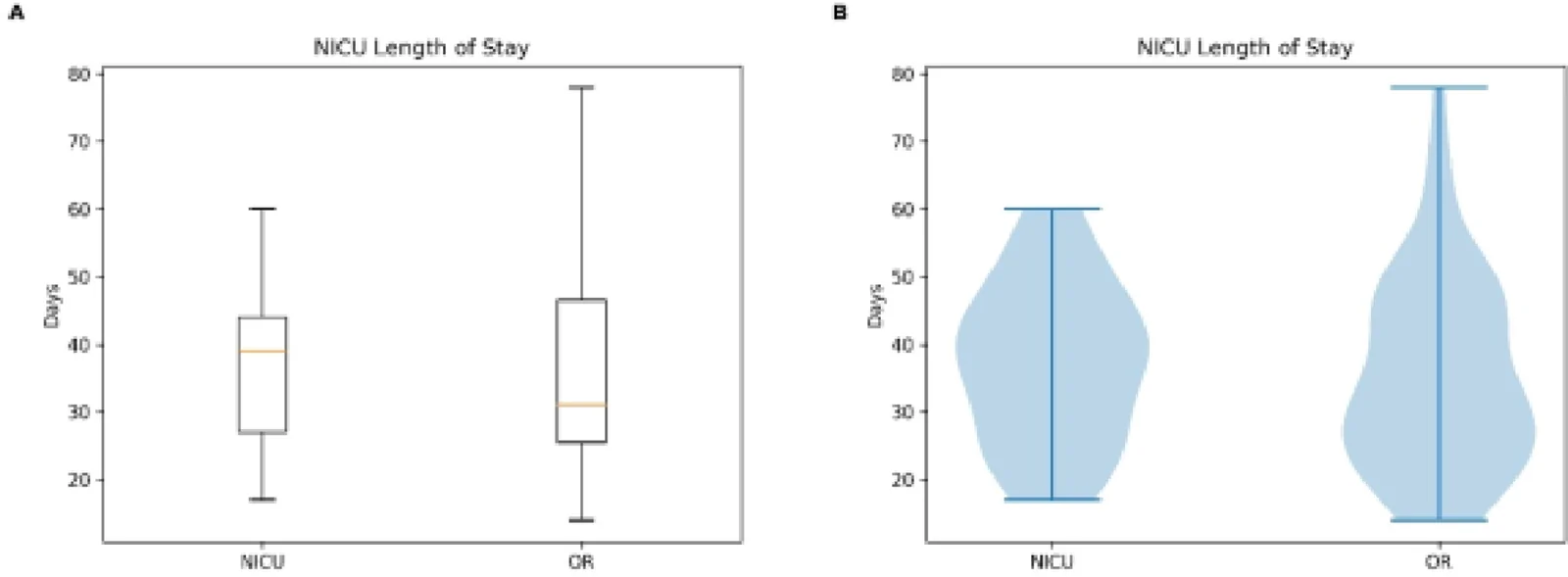
Comparison of NICU stay following surgical PDA ligation **(A)** Boxplot **(B)** Violin plot

The median duration of postoperative mechanical ventilation was 6 days (IQR 5–8.75) in the NICU group and 7 days (IQR 5–9) in the OR group (*p* = 0.940) (Fig. 3). The median postoperative NICU stay was 37.5 days (IQR 25.5–44.0) and 31 days (IQR 25.5–46.5), respectively (*p* = 0.650) (Fig. 4). PMA at NICU discharge was 36.1 weeks (IQR 34.9–37.7) in the NICU group and 36.7 weeks (IQR 34.4–38.4) in the OR group (*p* = 0.755).

Among infants weighing < 1000 g at surgery, eight were in the NICU group and 13 were in the OR group (Table 3). Infants in the OR group had a higher median weight at surgery than those in the NICU group (900 g [IQR 800–960] vs. 765 g [IQR 655–819], *p* = 0.019). PMA at surgery was also higher in the OR group (29.1 weeks [IQR 28.4–30.1] vs. 27.1 weeks [IQR 26.6–28.5], *p* = 0.027). Other baseline characteristics did not differ significantly.

Two infants in the NICU group died, compared with none in the OR group (*p* = 0.130). Both weighed < 1000 g, had preoperative acute renal dysfunction, and required vasoactive support before surgery. They died from progressive multiorgan failure on postoperative days 11 and 15, respectively. Neither death was associated with a documented intraoperative complication.

## Discussion

In this retrospective study, bedside PDA ligation using a standardized LAMT was technically feasible in preterm infants weighing ≤ 1500 g. All procedures were completed without conversion to sternotomy, intraoperative mortality, major bleeding, or vascular injury. The duration of postoperative mechanical ventilation, postoperative NICU stay, and postmenstrual age at NICU discharge did not differ significantly between the groups.

The main contribution of the study is the use of the same limited anterior approach for bedside and OR procedures. Previous studies have described PDA ligation in the NICU, but the surgical approaches were not uniform, and outcomes specifically related to LAMT were not consistently reported [12–14]. In the present study, all procedures were performed by a single surgeon using the same technique.

The anterior parasternal incision provides direct access to the ductus without posterolateral muscle division and with minimal lung retraction [9, 11]. Adequate exposure and secure clip placement were achieved in all cases, including infants weighing less than 1000 g. These findings support the technical applicability of LAMT in very small infants. However, the study did not include a conventional thoracotomy group and was not designed to compare the two approaches.

Avoiding transport is one of the main practical reasons for performing PDA ligation in the NICU. Extremely preterm infants frequently require invasive mechanical ventilation, thermal support, continuous monitoring, and multiple vascular lines. Transfer to and from the operating room may expose them to hypothermia, ventilatory instability, hemodynamic changes, and accidental displacement of the airway or vascular lines [13, 14, 17]. Bedside surgery allows the infant to remain in the established NICU environment without interruption of ongoing respiratory and supportive care.

Two infants in the OR group developed respiratory acidosis after transfer back to their referring hospitals. Both improved after adjustment of ventilator settings, and neither developed hypotension nor required vasoactive support. Detailed respiratory measurements before and after transfer were unavailable; therefore, these events cannot be attributed directly to transport. They nevertheless illustrate the limited respiratory reserve of mechanically ventilated preterm infants and the potential value of avoiding an additional transfer when bedside surgery is available.

No surgical site infection or postoperative sepsis was documented in either group during the first 7 postoperative days. Bedside procedures were performed in a designated area of the NICU using sterile instruments and equipment transferred from the OR. The number of patients was too small to compare infectious risk formally, but no early infectious complication specific to the bedside setting was identified.

No infant met the clinical definition of PLCS. However, serial echocardiographic measurements, detailed ventilator parameters and respiratory severity scores were not consistently available. The result should therefore be interpreted as the absence of clinically documented PLCS rather than proof that transient or subclinical cardiopulmonary changes did not occur. PLCS is related to the hemodynamic consequences of ductal closure and is not specific to the surgical approach or procedure location.

The subgroup of infants weighing less than 1000 g deserves particular caution. Infants in the OR group had a higher weight and greater postmenstrual age at surgery than those in the NICU group. This may indicate differences in maturity and clinical readiness for referral and intervention. The absence of mortality in the OR subgroup should therefore not be interpreted as evidence that surgery in the OR was safer.

Both deaths occurred in the NICU group among infants weighing less than 1000 g. Both had preoperative acute renal dysfunction and required vasoactive support before surgery. They died from progressive multiorgan failure on postoperative days 11 and 15, respectively, and neither death was associated with a documented intraoperative complication. Acute kidney injury in extremely preterm infants is associated with adverse outcomes and may occur as part of broader systemic and multiorgan dysfunction [16, 17]. These preoperative findings suggest that baseline illness severity was an important consideration when interpreting mortality.

The comparison between bedside and OR surgery was also limited by referral patterns. All infants in the NICU group were inborn, whereas all infants in the OR group were outborn and referred from external centers. Infants who underwent interfacility transfer may have represented a more stable population, while bedside surgery was available to infants already being treated in the institutional NICU, including those with greater preoperative instability. Because referral status and operative setting completely overlapped, their effects could not be separated. Consequently, the absence of statistically significant differences in short-term outcomes does not establish equivalence between the two settings.

Transcatheter PDA closure was available at our institution but was not performed in preterm infants during the study period. Transcatheter closure is increasingly used as an alternative to surgery, although it requires suitable anatomy, appropriate devices, and institutional expertise. Device embolization, vascular injury, and obstruction of the left pulmonary artery or aorta remain recognized concerns, particularly in very small infants [18–21]. The present study was not designed to compare surgery with transcatheter closure. Its findings instead describe LAMT as a surgical option when transcatheter treatment is not feasible or not routinely used.

This study has several limitations. It was retrospective, conducted at a single center, and included a relatively small number of patients and outcome events. Operative setting was completely associated with inborn/outborn status, and adjusted analysis was not possible. Detailed preoperative and postoperative ventilator parameters, respiratory severity scores, feeding status, pharmacological agents and number of treatment courses and standardized echocardiographic measurements were incomplete. These limitations restricted assessment of baseline illness severity and postoperative cardiopulmonary changes. Long-term outcomes, including vocal cord function, chest wall development, spinal deformity, and neurodevelopment, were also not assessed. Finally, all procedures were performed by a single surgeon. This improved technical consistency but may limit the generalizability of the results to other centers and surgical teams.

In conclusion, bedside PDA ligation through LAMT was technically feasible in selected preterm infants weighing ≤ 1500 g and allowed ductal closure without transport from the NICU. The approach was also successfully used in infants weighing less than 1000 g. However, the small sample size, limited clinical data, and complete overlap between operative setting and referral status preclude conclusions about equivalence or comparative safety between bedside and OR procedures.

**Table 1:** Baseline demographic and preoperative characteristics

	NICU group (*n* = 25)	OR group (*n* = 43)	*P* value
Gestational age (weeks), median (IQR)	27 (25–28)	26 (26–28)	0.94
Birth weight (g), median (IQR)	890 (735–1050)	840 (710–1022.5)	0.665
Female sex, n (%)	7 (28.0)	20 (46.5)	0.21
Weight at surgery (g), median (IQR)	1100 (875–1200)	1100 (980–1200)	0.37
Postnatal age at surgery (days), median (IQR)	23 (19–29)	26 (20–39)	0.17
PMA at surgery (weeks), median (IQR)	31.1 (28.7–32.3)	31.4 (29.6–33.0)	0.306
Preoperative acute renal dysfunction, n (%)	2 (8.0)	0 (0)	0.13
NEC, n (%)	3 (12.0)	5 (11.6)	> 0.99
IVH, n (%)	2 (8.0)	2 (4.7)	0.62
Preoperative vasoactive support, n (%)	2 (8.0)	0 (0)	0.13
Spontaneous intestinal perforation, n (%)	1 (4.0)	2 (4.7)	> 0.99

Values are expressed as median (interquartile range) or number (percentage). PMA, Postmenstrual age; NEC, Necrotizing enterocolitis; IVH, Intraventricular hemorrhage

**Table 2:** Postoperative outcomes and mortality

	NICU group (*n* = 25)	OR group (*n* = 43)	*P* value
Diaphragmatic paralysis, *n* (%)	1 (4.0)	1 (2.3)	-∗
Chylothorax, n (%)	0	1 (2.3)	-∗
Respiratory acidosis after transfer	0	2 (4.7)	-
Extubation time (days), median (IQR)	6 (5-8.75)	7 (5–9)	0.94
Post-ligation cardiac syndrome	-	-	-
Surgical site infection, n (%)	0	0	-
Postoperative sepsis (within 7 days), n (%)	0	0	-
Postoperative NICU stay (days), median (IQR)	37.5 (25.5–44.0)	31 (25.5–46.5)	0.65
Postmenstrual age at NICU discharge (weeks), median (IQR)	36.1 (34.9–37.7)	36.7 (34.4–38.4)	0.755
Mortality, n (%)	2 (8.0)	0	0.13

Values are expressed as median (interquartile range) or number (percentage). PMA, Postmenstrual age

∗ Statistical comparisons were not performed for selected variables because of very low event counts

**Table 3:** Infants Weighing < 1000 g Undergoing PDA Ligation

	NICU group (*n* = 8)	OR group (*n* = 13)	*P* value
Gestational age (weeks), median (IQR)	24.5 (24.0–25.0)	26.0 (25.0–27.0)	0.061
Birth weight (g), median (IQR)	660 (580–737.5)	690 (680–780)	0.179
Weight at surgery (g), median (IQR)	765 (655–819)	900 (800–960)	0.019
Postnatal age at surgery (days), median (IQR)	22.5 (20.5–24.5)	24.0 (22.0–28.0)	0.23
PMA at surgery (weeks), median (IQR)	27.1 (26.6–28.5)	29.1 (28.4–30.1)	0.027
Preoperative acute renal dysfunction, n (%)	2 (25.0)	0 (0)	0.13
NEC, n (%)	1 (12.5)	2 (15.4)	> 0.99
IVH, n (%)	1 (12.5)	1 (7.7)	> 0.99
Preoperative vasoactive support, n (%)	2 (25.0)	0 (0)	0.13
Spontaneous intestinal perforation, n (%)	0 (0)	1 (7.7)	> 0.99
Postoperative diaphragmatic paralysis	0	0	—
Chylothorax	0	0	—
PMA at NICU discharge (weeks), median (IQR)	34.9 (34.3–36.0)	34.7 (33.4–35.4)	0.805
Mortality, n (%)	2 (25.0)	0 (0)	0.13

Values are presented as median (interquartile range) or number (percentage). PMA, Postmenstrual age; NEC, Necrotizing enterocolitis; IVH, Intraventricular hemorrhage

## Data Availability

The datasets used and/or analyzed during the current study are available from the corresponding author on reasonable request.

## Abbreviations

PDA: Patent ductus arteriosus
hsPDA: Hemodynamically Significant PDA
LAMT: Left anterior mini-thoracotomy
PMA: Postmenstrual age
IQR: Interquartile range
IVH: Intraventricular hemorrhage
NEC: Necrotizing enterocolitis
NICU: Neonatal intensive care unit
OR: Operating room
